# Structure–Property Relationships in PEI/PET Polymer Blends: Morphological, Rheological, Thermal, Mechanical Behavior, and Electromagnetic Response

**DOI:** 10.3390/polym18121528

**Published:** 2026-06-19

**Authors:** Elshod Olmosovich Khakberdiev, Hülya Kaftelen Odabaşı, Akın Odabaşı, Selcuk Helhel, Qodirbek Nuridin ugli Berdinazarov, Nizomiddin Zokir ugli Dusiyorov, Nigmat Rustamovich Ashurov

**Affiliations:** 1 Institute of Polymer Chemistry and Physics, Uzbekistan Academy of Sciences, Tashkent 100174, Uzbekistan; profhaqberdiyev@gmail.com (E.O.K.); qodirberdinazarov@gmail.com (Q.N.u.B.); ndusiyorov@gmail.com (N.Z.u.D.); nigmat.ashurov@gmail.com (N.R.A.); 2 Department of Aircraft Maintenance and Repair, School of Civil Aviation, Fırat University, 23200 Elazığ, Turkey; 3 Department of Metallurgical and Materials Engineering, Engineering Faculty, Fırat University, 23200 Elazığ, Turkey; odabasia@firat.edu.tr; 4 Department of Electrical & Electronics Engineering, Engineering Faculty, Akdeniz University, 07058 Antalya, Turkey; selcukhelhel@akdeniz.edu.tr

**Keywords:** polymer blending, EMI shielding, mechanical properties, thermomechanical properties, rheological properties

## Abstract

In this study, twin screw extruded Polyetherimide (PEI)/Poly(ethylene terephthalate) (PET) polymer blends (90/10, 70/30, 50/50 *w*/*w*%) were investigated to elucidate the composition–property relationship governed by morphological, structural, rheological, thermomechanical, mechanical, and electromagnetic shielding (EMI) performance behavior. Among other polymer blends, the 70/30 blend exhibits superior thermomechanical stability with a significant glass transition temperature of 132.7 °C, where a robust confinement effect effectively restricts the mobility of PET chains. This morphology, characterized by a domain size of 562 nm, provides proof of concept for interface-driven attenuation, reaching a maximum EMI shielding effectiveness of 2.54 dB within the investigated blends. This performance is primarily governed by Maxwell–Wagner–Sillars polarization at the immiscible boundaries, alongside an optimized dielectric loss of tan δ ≈ 0.065. The design of these high-temperature PEI blends provides a proof of concept for interface-driven attenuation and demonstrates their potential for developing advanced EMI shielding matrices.

## 1. Introduction

The blending of two different polymers has been a subject of great interest in both academic and industrial fields, as it offers a combination of properties that differ from those exhibited by individual polymers and facilitates the development of multifunctional materials [1]. Thanks to their adjustable, multifunctional features, polymer blends can be used for a wide range of applications, including packaging, electronics, construction, aerospace, textiles, energy, and sustainable materials [2]. The blending of polymers enables the optimization of physical properties such as viscosity, crystallinity, and thermal stability through the presence of interfacial interactions. However, the compatibility of polymer blends and interfacial morphology are crucial factors in achieving optimal properties in polymer blends [3,4]. Polyetherimide (PEI) is an amorphous engineering polymer with high thermal stability, intrinsic flame retardancy and good mechanical properties at temperatures up to 200 °C, making it a suitable candidate for structural and electronic applications [5]. The presence of the bulky aromatic imide group in polyetherimide (PEI) provides high hardness, creep resistance, and thermal stability, while the flexible ether bond provides exceptional melt processability [6]. However, its high glass transition temperature (Tg) value of about 215 °C, and the resulting melt viscosity make processing difficult [3]. Poly(ethylene terephthalate) (PET) is a semi-crystalline polymer with a low Tg of approximately 78 °C and a melting temperature of around 255 °C [3,7]. Therefore, blending of PEI with a semi-crystalline poly(ethylene terephthalate) PET polymer with low Tg (78 °C) improves its processability, the solvent resistance, and mechanical properties of PEI and, can also reduce its cost [6]. In addition, the PEI-PET blend polymer benefits from the high glass transition temperature of PEI, which contributes thermal stability, while PET contributes dimensional stability and crystallinity [7,8]. Many researchers have investigated blends between PEI and PEI using various processing techniques, including melt processing and solution blending techniques, and studies have explored polymer blends rich in PET [6,9,10,11,12,13,14]. Mutlu et al. [13] aim to investigate the mechano-optical behavior and associated structural evolution during uniaxial stretching of melt-miscible PET/PEI (85/15, 75/25, 65/35 *w*/*w* %) blends near their glass transition temperature. Choi et al. [14] aim to understand how PEI content (PET/PEI = 100/0, 95/5, 90/10, 80/20, 70/30 *w*/*w*%) influences relaxation, crystallization kinetics, orientation development, and final mechanical/thermal properties. In another work of Choi et al. [6] focus on constrained annealing of pre-oriented PET/PEI films in a different context but similarly emphasizes kinetic transitions in mechano-optical response (e.g., regimes of birefringence vs. stress). While the earlier discussions often highlight real-time birefringence and mechano-optical aspects during deformation [6,13,14], Chen et al. [10] focus on thermal treatments (melting/annealing) and crystallization kinetics rather than in situ optical–mechanical coupling. The crystallization of the PET/PEI blends was dramatically slowed by PEI contents higher than 20 wt.%, as reported by Ruvolo and Borros [9], who studied the miscibility, crystallization, and melting behavior in blends of virgin and recycled PET with PEI (0, 5, 10, 20, 25, 35 and 50 *w*/*w*%) using DSC. In another study [11] involving PEI/PET blends (containing 100, 80, 60, 40, 20, and 0 wt.% PET), the effect of the composition on thermal structural behavior, namely the glass transition temperature and the crystallization kinetics of the PET polymer, was investigated. It was observed that increasing PEI content suppresses PET crystallization, altering the crystallization kinetics and final morphology of PET/PEI polymer blends (40/60, 55/45, 70/30, 100/0 *w*/*w*%) [10,11]. Although literature studies have comprehensively examined PET/PEI polymer blend systems [6,10,11,12,13], existing studies have primarily focused on miscibility behavior and PET-rich compositions, emphasizing thermal stability, crystallization kinetics, and mechano-optical properties. In contrast, researchers have scarcely studied PEI-rich PET/PEI blends despite inherent thermal and mechanical advantages of PEI and the potential of phase-separated morphologies to provide functional performance in advanced applications. This study aims to address the existing gap in the literature by focusing on PEI-rich PEI/PET polymer blends and thoroughly investigating their structural, morphological, rheological, thermomechanical, and mechanical properties to identify compositions that can serve as a potential polymer matrix for EMI shielding applications. In this study, PEI-rich PET/PEI polymer blends were systematically investigated to elucidate the role of composition-induced immiscibility in morphology development and physical–mechanical properties. The presence of phase-separated structures in immiscible blends is expected to affect interface properties and melting behavior. These factors are critical in determining the formation of conductive networks and electromagnetic attenuation mechanisms.

## 2. Experimental

### 2.1. Materials and Preparation of Polymer Blends

In this work, polyetherimide (PEI) (Ultem-1000) was purchased from SABIC (Riyadh, Saudi Arabia). The PET waste bottle feedstock was sourced from post-consumer water bottles obtained from a single source (Aquafina, PepsiCo, NY, USA), which helped limit the variability associated with mixed feedstock streams. Prior to processing, the PET chips were thoroughly washed and dried to remove surface impurities and moisture. As the material originates from food-grade applications, the level of contamination is expected to be minimal. The PET investigated here had an intrinsic viscosity of approximately 0.70–0.85 dL/g [15,16]. The bottle-grade PET typically exhibits an average molecular weight in the range of 24,000–36,000 g·mol^−1^ [17]. The melt flow index (MFI) of the PET was measured as 69 (at 280 °C, 2.16 kg) according to ISO 1133 [18]. Regarding the thermal history of the materials, melt-compounding was performed at 240 °C, followed by rapid quenching. This specific processing route was selected to minimize differences in thermal history and to establish a standardized morphological baseline across all samples. For the characterization of the blends, since the ISO standard [18] prescribes different testing conditions for PEI and PET, all measurements for the blends were conducted under the conditions specified for PEI (340 °C/5 kg), as it constitutes the continuous matrix phase. It should be noted that pure PET could not be reliably tested at these higher conditions (340 °C/5 kg) due to its extremely low melt viscosity and the accelerated risk of thermal degradation and chain scission at such elevated temperatures [19,20]. However, the results presented in Table 1 reveal a significant trend: the MFI increased from 65 g/10 min for pure PEI to 358 g/10 min for the PEI/PET 50/50 polymer blend. This substantial increase (over 450%) clearly demonstrates that recycled PET acts as a potent rheology modifier and processing aid within the PEI matrix, facilitating molecular mobility and significantly enhancing the melt flow of the composite [21]. Before the polymer blends were prepared, PEI and PET polymers were dried in a vacuum oven at 110–130 °C for 24 h under a pressure of 50–100 Pa (≈0.5–1.0 mbar) to effectively remove residual moisture and prevent hydrolytic degradation of PET during extrusion. Due to the lack of high-precision moisture analyzers, the residual moisture content could not be directly measured after drying; this remains a limitation of this study. Although the residual moisture content was not directly measured, the applied conditions are consistent with commonly reported protocols and are generally sufficient to reduce moisture levels below the critical threshold (~0.02%) [19,22]. However, to minimize the risk of hydrolytic degradation, which could significantly impair the mechanical and dielectric performance of polymer composites [23], the drying protocol was strictly followed in accordance with established standards in the literature [19,22].

**Figure 1:**
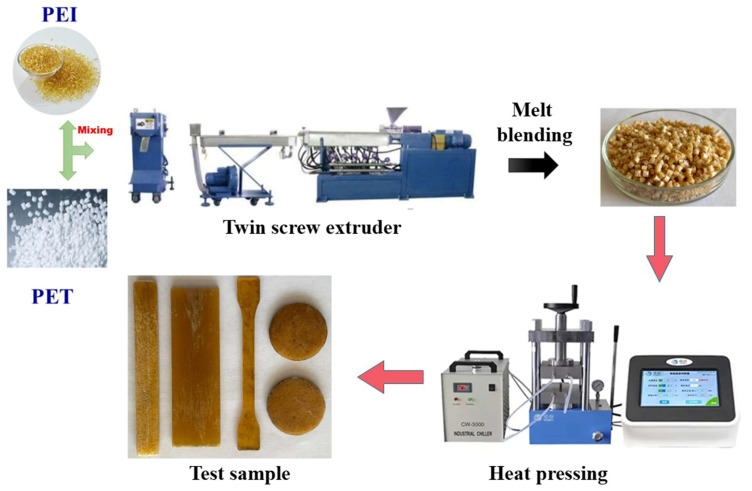
A detailed depiction of the PEI/PET fabrication process.

The matrix polymer materials were prepared on the basis of pure PEI, PET, and PEI/PET blends with weight ratios of 90/10, 70/30, and 50/50, were melt-compounded using a co-rotating twin-screw extruder (KTE-20A Lab, Nanjing Kerke Extrusion Equipment Co., Nanjing, China) with a screw diameter of 21.7 mm and an L/D ratio of 40. The extrusion process was conducted at a temperature of 240 ± 5 °C, with the screw speed and feed rate maintained at 50 rpm and 5 kg/h, respectively. Under these stabilized operational conditions, the average residence time within the barrel was approximately 1.5–2 min. This configuration was strategically selected to ensure optimal distributive and dispersive mixing of the constituents while mitigating the risk of thermomechanical degradation. Following the extrusion stage, the extrudates were pelletized via granulation. The resulting granules were subsequently processed through compression molding at a temperature of 250 °C for a duration of 2 min under a constant pressure of 2.5 MPa (25 bar). Upon completion of the molding cycle, the specimens were subjected to rapid pressure-assisted water quenching to ensure a high cooling rate. This protocol was strategically implemented to suppress post-crystallization phenomena and to preserve the specific morphological characteristics developed during the melt-processing stage. Hereafter, neat PEI and recycled PET, as well as PEI polymer blends containing 10, 30, and 50 wt.% PET, were referred to as PEI100, PET100 and PEI/PET 90/10, PEI/PET 70/30 and PEI/PET 50/50, respectively. Figure 1 shows the schematic presentation of PEI/PET polymer preparation.

### 2.2. Characterizations

The infrared spectral properties of PEI/PET polymer blends were analyzed using an Inventio-S IR Fourier device (Bruker, Karlsruhe, Germany) with attenuated total reflectance (ATR) technology, in the 400–4000 cm^−1^ frequency range, with a resolution of 2 cm^−1^. X-ray diffraction (XRD) is used to analyze the degree of crystallinity and structural changes. X-ray diffraction analyses of the polymer blends were carried out using a Rigaku Miniflex 600 (Japanese) instrument (Tokyo, Japan) with 40 kV voltage, 15 mA current, and 0.02° step. The average crystallite size (D) was estimated using the Debye-Scherrer equation [24]:

(1) $$D=\frac{K\lambda }{\beta cos\theta }$$

where D average crystallite size, K is shape factor (the value of K was taken as 0.9) [16], λ is X-ray wavelength, β is full width at half maximum of the diffraction peaks (in radians, corrected for instrumental broadening), and θ is the Bragg angle.

Thermal stability of polymer blends was carried out using thermogravimetric analysis (TGA, Selb, Germany; Linseis PT1610a) under an inert atmosphere. Measurements were taken at a heating rate of 10 °C/min in the temperature range of 20–800 °C, and the second scan data were used. The thermo-mechanical properties of the PEI/PET blends were analyzed using a NETZSCH DMA 242E (Selb, Germany) in a three-point bending configuration. Standard rectangular specimens (52 mm long,13 mm wide, 3 mm thick) [25] were heated from 25 °C to 160 °C at a ramp rate of 2 °C/min under a nitrogen flow. Storage modulus, and damping factor (tan δ) were recorded as a function of temperature. Glass transition temperature (Tg) of the PEI/PET blends was determined from the peak temperature of tan δ curve, representing the maximum molecular damping of the polymer chains. The thermal transitions of neat recycled PET and pure PEI were characterized from 40 °C to 240 °C at a heating rate of 10 °C/min using a differential scanning calorimeter under a constant nitrogen purge (20 mL/min) (DSC, Perkin Elmer Sapphire, Waltham, MA, USA). The surface morphological properties of 0.5 mm-thick, 1 mm-wide, plate-shaped samples prepared from polymer blends were analyzed using an atomic force microscope (AFM, Agilent 5500 scanning probe microscope, Santa Clara, CA, USA). Silicon cantilevers with a stiffness of 9.5 N/m and a frequency of 145 kHz were used in the study. The maximum scanning area in the X and Y dimensions in AFM is 15 × 15 μm^2^, and 1 μm in the Z dimension.

The rheological properties of the samples were measured in time scan, strain scan, and frequency scan modes. In this case, results were obtained where complex viscosity, dynamic modulus, and loss modulus values were plotted against frequency, graphically shown on logarithmic coordinate axes. Disc samples with a diameter of 25 mm were prepared from the dried granules using a thermopress at 250 °C temperature and 50 bar (5 MPa) pressure, and these samples were stored at 80 °C in a vacuum until rheological measurements were performed. A dynamic oscillatory stress-controlled rotational test was performed in parallel plate geometry mode using a TA Instruments (Anton Paar MCR 92 rheometer, Graz, Austria). The test was carried out at 240 °C, from 0.1 to 100 Hz, and with 1% of strain determined from the amplitude sweeps test.

The mechanical properties of polymers were evaluated in terms of elongation at break, tensile strength, and elastic modulus using the Shimadzu AG-X PLUS (Kyoto, Japan) tensile test instrument. Samples made from polymer blends were shaped in accordance with the international standard ASTM D638-99 [26]. In order to measure elastic modulus (E), a tensile speed of 1 mm/min was applied up to 0.3% strain. After that, the tensile speed was immediately increased to 20 mm/min to assess the tensile strength (σ) and elongation at break (ε). Tensile test specimens of PEI/PET polymer blends were prepared using an injection molding (Zamak Mercator, Skawina, Poland) at 250 °C temperature and 5 bar pressure. To ensure statistical significance and representativeness of the tensile test results, five specimens were systematically collected from different regions of the extruded material for each composition. The mechanical data are reported as the mean standard deviation. The fracture surface morphology of the PEI/PET 70/30 specimens after tensile testing was examined using a scanning electron microscope (SEM, Scios 2 DualBeam) (Thermo Scientific, Waltham, MA, USA). The EMI shielding effectiveness (SE) in the 8–12 GHz (X-band) frequency range was measured using an Agilent N5247A vector network analyzer (VNA, Santa Clara, CA, USA) connected to a rectangular waveguide. The EMI shielding data presented is the average of 10 different measurement campaigns using a polymer sheet of 10 mm × 22 mm × 3 mm. The dielectric permittivity values (ε′ and ε″) of the composites were obtained from the measured S_11_ and S_21_ using the robust Nicolson–Ross–Weir (NRW) method, the standard analytical approach for waveguide-based material characterization. Further details regarding the EMI shielding measurements have been described in our previous studies [27,28].

## 3. Results and Discussions

### 3.1. Structural Properties

**Figure 2:**
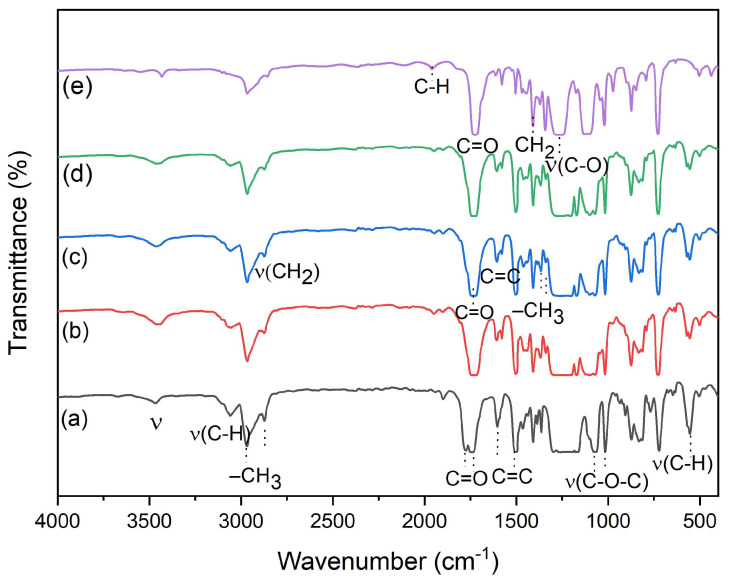
FTIR spectrum of (a) PEI 100, (b) PEI/PET 90/10, (c) PEI/PET 70/30, (d) PEI/PET 50/50, and (e) PET 100.

FTIR analysis was carried out to determine the functional groups of polymers and to evaluate the degree of chemical interaction between the components of a mixture. Figure 2 shows the FTIR spectra of the pure PEI, pure PET, and PEI/PET polymer blends prepared at different mass ratios.

In the fingerprint region (600–1500 cm^−1^), absorption bands corresponding to the vibrations of aromatic and aliphatic moieties are observed, while in the valence vibration region (1500–3500 cm^−1^), specific bands for carbonyl (C=O) and ether groups are detected. The pure PEI spectrum (Figure 2a) exhibits a characteristic peak at 1898 cm^−1^ related to the imide carbonyl groups. The band at 3467 cm^−1^ is assigned to the overtone of the intense imide carbonyl stretching vibrations or the presence of trace amounts of absorbed moisture [29]. The peaks at 2968 cm^−1^ and 2873 cm^−1^ correspond to the asymmetric and symmetric stretching vibrations of the methyl (CH_3_) groups, respectively [29]. Additionally, a weak absorption at 3060 cm^−1^ is attributed to the C-H stretching vibrations of the aromatic rings [29,30]. The imide group is further characterized by asymmetric and symmetric C=O stretching at 1775 cm^−1^ and 1736 cm^−1^, respectively [29]. Aromatic ring (C=C) vibrations are observed as a weak peak at 1603 cm^−1^ and an intense peak at 1504 cm^−1^. The bands at 1464 cm^−1^ and 1436 cm^−1^ are assigned to the asymmetric and symmetric scissoring vibrations of the methyl group, accompanied by CH_3_ scissoring at 1464 cm^−1^ and 1436 cm^−1^ [29]. Furthermore, the band at 1408 cm^−1^ is associated with the aromatic ring skeletal vibrations, while the moderate-intensity bands at 1386 cm^−1^ and 1364 cm^−1^ correspond to the in-plane deformation vibrations of the CH_3_ groups [29]. The intense peaks at 1298 cm^−1^ and 1241 cm^−1^ are related to the asymmetric vibrations of the ether bonds [29]. During the preparation of PEI/PET-based polymer blends, notable changes in the absorption bands were observed, indicating intermolecular interactions between the functional groups of PEI and PET. As can be seen from the experimental data, the characteristic bands of the initial samples are preserved in the PEI/PET 90/10 blend (Figure 2b), albeit with slight shifts compared to the pure components. The weak absorption band at 3467 cm^−1^ in the pure PEI spectrum shifts to 3447 cm^−1^ in the blends (see Figure 2b–d). This shift is attributed either to an overtone of the intense imide carbonyl stretching vibrations or to trace amounts of absorbed moisture. This suggests a modification in the hydrogen bonding environment or dipole–dipole interactions upon blending [29]. The absorption line at 1898 cm^−1^ in the pure PEI spectrum, associated with the imide carbonyl groups, splits into two bands in the PEI/PET blends (1948–1900 cm^−1^), indicating changes in the carbonyl environment due to the proximity of PET chains. Instead of the separate peaks at 1775 cm^−1^ and 1736 cm^−1^, a consolidated band at 1728 cm^−1^ is observed, corresponding to the stretching vibrations of the imide C=O groups. The broadening and a slight shift toward lower wavenumbers of the C=O band observed in the FTIR data of PEI/PET polymer blends (Figure 2b–d) suggest composition-dependent intermolecular interactions between PEI and PET chains. Furthermore, the appearance of a moderate doublet in the 1607–1579 cm^−1^ range indicates that stacking between the aromatic rings of both polymers likely contributes to the stability of the PEI/PET blends. The distinctive peaks for the pure PET sample (Figure 2e) at 2967 cm^−1^ and 2856 cm^−1^ are associated with the asymmetric and symmetric stretching vibrations of the methylene groups, respectively [31]. A shoulder-shaped absorption band at 3060 cm^−1^ corresponds to the C-H stretching of the aromatic ring. The band at 1960 cm^−1^ is related to the summation/overtone vibrations characteristic of para-substituted aromatic rings [31]. The intense absorption at 1727 cm^−1^ is assigned to the stretching vibrations of the C=O (carbonyl) group. Additionally, weak peaks observed at 1613 cm^−1^, 1579 cm^−1^, and 1504 cm^−1^ correspond to the C = C stretching vibrations of the aromatic ring. In the PET spectrum, the doublet peaks at 1470 cm^−1^ and 1445 cm^−1^ correspond to the asymmetric and symmetric shear vibrations of the methylene groups, respectively. In addition, a medium-intensity peak is observed at 1409 cm^−1^, corresponding to the aromatic ring vibrations [32,33]. The asymmetric and symmetric vibrations of methylene groups are observed at wave numbers of 1371 cm^−1^ and 1342 cm^−1^, respectively. The broad and very intense band at 1270 cm^−1^ corresponds to the asymmetric stretching vibrations of the ester C-O bonds. Symmetric vibrations of these bonds are observed in the range between 1174 cm^−1^ and 973 cm^−1^, depending on their trans or gauche conformational states. The peak at 727 cm^−1^ is associated with the C-H out-of-plane bending of the para-substituted aromatic rings, reflecting the deformation of carbon-hydrogen bonds within the terephthalate unit [32]. FTIR results indicate that the absorption bands in the infrared spectrum undergo significant changes due to the intermolecular interactions between the functional groups in PEI/PET polymer blends, which directly affect the physicochemical properties of the resulting materials. These FTIR results confirm that intermolecular interactions between functional groups directly influence the physicochemical properties of the resulting polymer blends.

**Figure 3:**
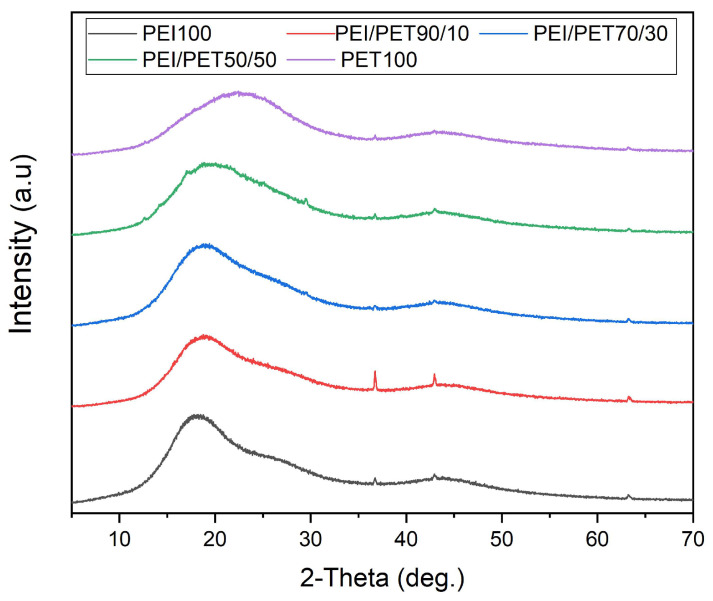
XRD patterns of PEI, PET, and their blends.

X-ray diffraction (XRD) patterns were analyzed to investigate changes in crystalline structure, peak intensity, and peak broadening as a function of blend composition. X-ray structural studies have shown that the intensity and width of the diffraction peaks of PEI/PET composite samples vary depending on the proportions of the two materials in the sample (Figure 3).

X-ray diffraction analysis revealed crystallographic reflections at 2θ = 12.58°, 14.24°, 17.04°, 19.38°, 25.08°, 29.52°, 36.75°, 39.34° and 42.49° in the PEI/PET 50/50 composite diffraction plot. The maximum point with the highest intensity was located at 2θ = 19.38°, which corresponds to the crystallographic reflection (012) plane. At the same time, a much lower intensity of reflection corresponding to the (013) plane was observed at 2θ = 43.93° (see Figure 3). The highest intensity maximum for the initial PET, however, is observed at 2θ = 22.99°, which corresponds to the (111) crystallographic reflection. A low-intensity crystalline reflection was also recorded at 2θ = 43.91°, corresponding to the (214) plane. X-ray diffraction analysis revealed that the PET sample comprises crystals with a triclinic structure, consistent with existing literature [34]. However, the unit cell parameters differ. A small proportion (2%) of the PET crystals exhibit triclinic structure and are characterized by the following lattice parameters: a = 4.59 Å, b = 5.90 Å, c = 11.05 Å, α = 96.6°, β = 120°, γ = 111°. Meanwhile, the majority of the PET sample (64%) exhibits the following lattice parameters: a = 4.18 Å, b = 5.56 Å, c = 13.50 Å, α = 96.6°, β = 118.9°, γ = 112°.

A large amorphous halo is observed in the X-ray patterns of initial PEI and polymer blends in various ratios, indicating that there is no long-range order in the structure of these samples. This is presumably due to the high PEI content in the samples. The XRD pattern of neat PEI exhibits a broad diffraction halo centered around 2θ = 18–22°, which is characteristic of its amorphous structure. Based on X-ray analysis of these polymer crystals, their maximum location can be estimated. In our case, the amorphous halo has a bimodal shape, and its maxima are observed at 2θ = 19.8° and 44°. These maxima may be related to the helical structure of the system.

Notably, as the PET content increases, a slight shift and intensity variation in the main diffraction halo were observed, indicating changes in chain packing density. To further elucidate the structural evolution, the crystalline characteristics of the PEI/PET 70/30 blend were investigated using XRD deconvolution with Lorentz line-shape fitting (refer to Supplementary Figure S1 and corresponding table for detailed fitting parameters). The crystalline reflections in the XRD analysis were successfully resolved from the amorphous background with high precision (Adj. R^2^ = 0.99585), providing a robust basis for calculating the lattice parameters [35]. These resolved peaks are consistent with the triclinic crystal system of PET, where the highest intensity crystalline reflection was identified in the plane [36,37]. The crystallite size in this specific direction was estimated to be 6.9 nm using the Debye–Scherrer equation [24]. Based on the refined peak positions, the PEI/PET (70/30) sample exhibits a triclinic unit cell with the following lattice parameters: a = 6.04 Å, b = 7.35 Å, c = 10.59 Å, α = 74.57°, β = 72.52°, and γ = 81.54°. It should be noted that due to the predominantly amorphous nature of PEI/PET blends, these calculated crystallite sizes represent approximate values reflecting local molecular ordering within the polymer matrix [37]. The highest crystal reflections in the diffractograms of the PEI/PET (90/10) sample were observed at 2θ = 9.6°, 16.4°, 18.7°, 24.7°, 28.2°, 31.8°, 36.7°, 41.1°, 42.9°, 44.5°, 56.1° and 63.2°. Crystallite growth was observed in directions [122], [123], and [313] in this composition, at 2θ = 36.7°, 42.9°, and 63.2°, respectively. As shown in Figure 3, experimental data indicate that the presence of a small amount of PET increases the intensity of PEI’s crystalline reflections, particularly at 2θ = 36.7°, 42.9°, and 63.2°. This phenomenon is due to the structures of PEI and PET leading to an increase in crystalline reflection intensity, resulting in an increase in regularized areas oriented in this stoichiometric ratio.

### 3.2. Rheological Properties

Rheometry analyses help determine the viscosity level and flow mechanics of the polymer blends. A detailed study of these parameters allows us to determine the optimal technological conditions for the extrusion process and, as a result, to produce high-quality polymer compositions. The rheological properties of PEI and PET blends were investigated using oscillatory shear measurements. Frequency sweeps were performed to evaluate the viscoelastic response of the blends and to analyze the effect of blend composition on their microstructure and miscibility.

**Figure 4:**
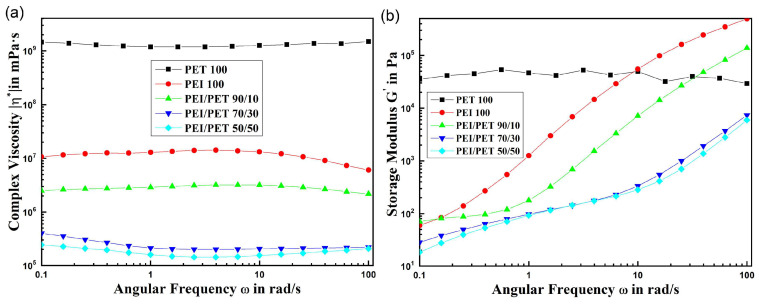
(**a**) Complex viscosity and (**b**) storage modulus of neat PEI, PET and PEI/PET blends.

Figure 4a shows the complex viscosity (η*) versus angular frequency (ω) curves, which demonstrate shear-thinning behavior for both neat polymers (PEI100 and PET100) and their blends. As shown in this figure, the complex viscosity of the PEI/PET blends decreases significantly, falling two to four orders of magnitude below that of the neat components across the entire frequency range. This phenomenon, known as negative deviation behavior, is an indicator of highly immiscible polymer systems, in which interfacial slippage and chain disentanglement occur at the phase boundaries [38,39,40]. Notably, the neat recycled PET exhibits remarkably high complex viscosity values (up to 10^9^ mPa·s), indicating that the molecular weight remained stable during extrusion due to the rigorous vacuum drying protocol (110–130 °C for 24 h) (Figure 4a). This high baseline confirms that the significant decrease in viscosity observed in the PEI/PET 50/50 and 70/30 blends is not a result of polymer degradation or hydrolytic chain scission [16]. Instead, this rheological behavior is attributed to the phase morphology of the blends and the specific interactions between the PET and PEI phases [22,41]. Ultimately, this behavior confirms the highly immiscible nature of the PEI/PET blend system and its significantly reduced interfacial adhesion [42,43].

In Figure 4b, the storage modulus (G′) of the neat PEI and PET polymers and their blends is presented. While neat PET (PET 100) exhibits a high and stable elastic response (10^4^–10^5^ Pa), the blends show a dramatic non-additive decrease in G′, falling approximately two orders of magnitude below both neat components. This significant negative deviating elasticity indicates poor interfacial coupling and a reduced ability to store elastic energy. This is because the immiscible boundaries facilitate interfacial slippage instead of stress transfer [40]. The absence of a low-frequency elastic shoulder, which would normally be expected for compatible droplets, further confirms the weakly coupled morphology of PEI/PET blends [44]. The significant difference between the neat PET baseline and the blends conclusively proves that this rheological drop is a morphological consequence of the immiscible interface, rather than thermal chain scission [16,45].

### 3.3. Atomic Force Microscopy (AFM) Analysis

**Figure 5:**
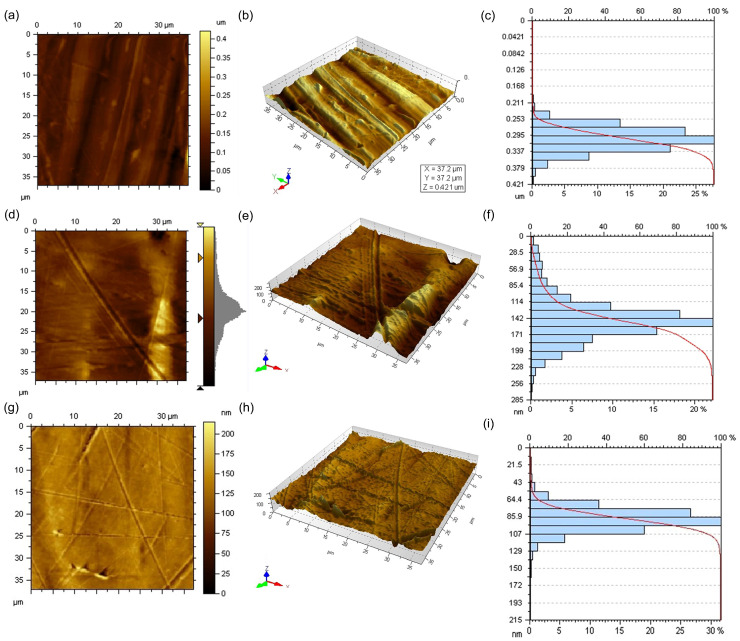
Atomic force microscopy (AFM) images and corresponding height histogram, 3D atomic force microscopy (AFM) topographical maps of (**a**–**c**) PEI/PET (90/10), (**d**–**f**) PEI/PET (70/30), and (**g**–**i**) PEI/PET (50/50) polymer blends.

AFM was utilized to examine the surface morphology of PEI/PET blend films, providing detailed information on phase distribution, interfacial characteristics, and the evolution of surface roughness. These analyses are crucial for understanding the microstructural features and their dependence on blend composition. AFM analysis (Figure 5a) of PEI/PET 90/10 blend revealed a rigid and relatively rough and heterogeneous surface, with an average roughness of ~253 nm and peak height reaching up to 420 nm.

Increasing PEI content reduced PET dispersion, resulting in pronounced phase separation and abrupt variations in surface relief. This reflects the contribution of PET-rich domains embedded within the PEI matrix. Histogram analysis further confirmed a low PET fraction (5–15%), indicating a fine but uneven distribution within the matrix. The elongated ridge-like patterns distributed along the scan direction in the AFM image likely originated from phase-separated domains in polymer blends. The microstructural heterogeneity of the PEI/PET system and the presence of interfacial properties further support the asymmetric shape of the distribution. The surface morphology of PEI/PET 70/30 polymer blends, given in Figure 5b, indicates a pronounced change in the surface topography with increasing PET content (30 wt.%) compared to the PEI/PET 90/10 polymer blends. This is characterized by deep valleys and prominent ridge-like structures. According to the histogram, the surface roughness reaching approximately 142 nm with a peak height of 285 nm demonstrates the strong influence of PET content on the surface morphology. The dispersed PET domains accounted for 10–30%, reflecting reduced PET content and a dominant PEI phase. The three-dimensional (3D) AFM scan image further revealed a non-uniform surface profile with interfacial cracks and distinct phase boundaries. The AFM surface morphology of PEI/PET 50/50 polymer blends, as represented in Figure 5c, exhibited an average roughness of ~86 nm and a peak height of ~215 nm. The height distribution displays a narrower and more symmetric distribution compared to the PEI/PET 70/30 polymer blend composition. The surface morphology appeared moderately smooth, indicating partial interdiffusion between PEI and PET phases at equal blend ratios. However, localized depressions and cracks suggested incomplete phase compatibility. Histogram analysis revealed a more balanced PET/PEI distribution (20–45%), yet interfacial inhomogeneities remained evident. The domain size (fibril widths) of the PEI/PET polymer blends was calculated from the phase images obtained using atomic force microscopy (AFM) and analyzed using Image J 1.53m software [46]. The width of the domains (fibrils) in PEI/PET polymer blends was measured directly rather than using area-based approximations. This linear measurement method allows for a more accurate representation of the phase dimensions in commonly observed elongated or fibrillar structures. For each sample, at least 100 individual measurements were performed on SEM micrographs to ensure both the statistical significance and representativeness of the data. The statistical distribution of the PET domain widths in the PEI matrix and the corresponding Gaussian fitting parameters for all blend compositions were provided in the Supplementary Information (Figures S2 and S3). Quantitative analysis revealed that the mean domain widths (x_C_) (see Figure S3) for the PEI/PET binary blends were about 1003 ± 27.12 nm for the 50/50 ratio, 562.5 ± 12.34 nm for the 70/30 ratio, and 928.4 ± 21.63 nm for the 90/10 ratio. The 70/30 composition exhibited the most refined phase dispersion, creating a substantial interfacial area that is critical for enhancing Maxwell–Wagner–Sillars polarization [47], whereas significant domain coarsening was observed at both higher and lower PET concentrations. These values, obtained from domain size (fibril width) measurements per sample using Image J, demonstrate a non-linear relationship between the component ratio and the resulting morphology. These results confirm that the PEI/PET ratio critically governs the degree of phase separation and morphological uniformity, with the 70/30 ratio providing an optimal balance between refined domain size and interfacial connectivity, unlike the coarser structures observed at higher or lower PET fractions [48].

### 3.4. Thermal Properties

**Figure 6:**
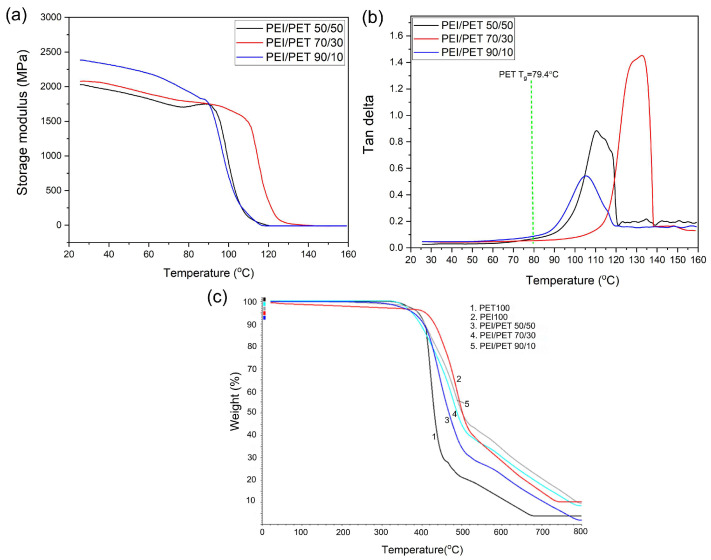
Dynamic mechanical analysis of PEI/PET blends at various weight ratios (90/10, 50/50, and 70/30): (**a**) Storage modulus (E′), (**b**) damping factor (tan δ), and (**c**) TGA thermograms of neat PEI, PET, and PEI/PET polymer blends. The vertical dashed line shown in green in Figure 6b indicates the Tg value for recycled PET (PET 100).

The thermo-mechanical analysis (DMA) and thermogravimetric analysis (TGA) of the PEI/PET blends were investigated. Figure 6a illustrates the storage modulus (E′) as a function of temperature, providing insight into the stiffness and load-bearing capacity of the composites. Figure 6b shows the tan δ curves used to identify the glass transition temperatures. Figure 6c shows the thermogravimetric analysis (TGA) of neat PEI and PET polymers and their blends with different compositions (90/10, 70/30, and 50/50 *w*/*w*%) used to identify their thermal decomposition behavior.

The storage modulus (E′) curves (Figure 6a) show a sharp decline typical of the glass transition region, which corresponds to the transition from a glassy to a rubbery state. The onset temperatures for this transition were determined to be 91.5 °C, 94.3 °C, and 112.1 °C for the PEI/PET 90/10, PEI/PET 50/50, and PEI/PET 70/30 blends, respectively. At these points, the storage modulus values were 354 MPa, 289 MPa, and 222 MPa, respectively. The increase in onset temperature with higher PEI content, particularly for the PEI/PET 70/30 blend, suggests that the high-PEI phase significantly restricts the thermal initiation of PET chain mobility. The catastrophic drop in modulus in the 90/10 blend following the PET transition at 105.03 °C is primarily caused by the suppression of PET crystallization within the dominant amorphous PEI matrix [22]. This effect is compounded by partial miscibility, whereby dissolved PET chains act as a plasticizer that destabilizes the PEI network’s load-bearing capacity during segmental relaxation [38]. Consequently, the PEI/PET 90/10 system lacks the crystalline confinement and phase-to-phase reinforcement observed in the 70/30 blend, resulting in a rapid loss of structural integrity [13]. The glass transition temperatures (Tg) derived from the peak maxima of the tan δ curve show a distinct dependency on the blend composition. The values were found to be 105.03 °C for PEI/PET 90/10, 110.52 °C for PEI/PET 50/50, and 132.7 °C for PEI/PET 70/30. To provide context for these shifts, the Tg values of the neat components were determined using DSC (see Figure S4 in the Supplementary Material). The neat PET exhibited a Tg of 79.4 °C, and the neat PEI showed a Tg of 217 °C. The vertical dashed line in Figure 6b indicates the Tg of neat recycled PET (79.4 °C) as shown in the DSC graph in the Supplementary Material (Figure S4). The primary relaxation peaks of all blends are significantly shifted to higher temperatures. Notably, the Tg value in the PEI/PET 70/30 blend shifted to 132.7 °C, representing an increase of approximately 53 °C compared to the neat PET baseline. This substantial upward shift is attributed to the ‘confinement effect,’ where the segmental mobility of PET chains is severely restricted by the surrounding rigid PEI matrix [16,39,49]. The presence of the PEI phase, which remains in a glassy state up to 217 °C, effectively hinders the large-scale molecular relaxation of the PET fraction [20].

A notable observation in Figure 6b supporting this phenomenon is the variation in peak height. The PEI/PET 70/30 blend (red curve) exhibits the highest tan δ peak (~1.45), suggesting high energy dissipation and molecular friction during the PET phase transition. Furthermore, the curve for the PEI/PET 70/30 blend exhibits a distinct shoulder between 110 °C and 125 °C, preceding the main peak at 132.7 °C. This dual feature indicates a non-mixing phase structure. It represents a heterogeneous relaxation spectrum: the low-temperature shoulder corresponds to ‘bulk-like’ PET regions, while the peak at 132.7 °C represents the PET fraction in close interaction with, or largely confined by, the PEI-rich phase [39]. Similarly, the PEI/PET 50/50 blend (black curve) shows a high-temperature tail, representing a ‘constrained’ PET fraction at the phase interface between the PET and PEI phases [39]. By contrast, the 90/10 blend shows the lowest peak intensity (~0.55) due to the low volume fraction of the PET phase and its crystallization being suppressed by the surrounding amorphous PEI matrix [13,16]. Unlike the other blends, the 90/10 composition does not show a second distinct peak for the PEI phase above 210 °C, the tan δ curve of the PEI/PET 90/10 blend does not show a second distinct peak for the neat PEI phase, which is typically expected to occur at temperatures above 210 °C [50]. Analysis of the TGA curves revealed that the thermal stability of the blends is significantly enhanced by the high aromatic content of the PEI matrix. While pure PET undergoes rapid thermal decomposition between 390–400 °C, the addition of PEI effectively shifts the degradation onset toward higher temperatures, with the PEI/PET 90/10 blend exhibiting the highest overall chemical stability. The two-step degradation observed in the PEI/PET 50/50 and PEI/PET 70/30 blends confirms their immiscible phase structure, where each peak corresponds to the distinct decomposition of PET and PEI phases [47]. Although the PEI/PET 90/10 blend shows the highest chemical resistance in TGA, its early mechanical softening in DMA highlights the difference between chemical bond strength and the physical plasticization effect of the amorphous PET fraction [13]. In other words, the absence of a second in the DMA is due to the mechanical softening of the PET phase, rather than phase miscibility. This synergy suggests that, although PEI determines the ultimate decomposition limits, phase morphology—specifically the confinement effect observed at a PEI/PET 70/30 ratio—is the main factor influencing high-temperature mechanical performance.

### 3.5. Mechanical Properties

**Figure 7:**
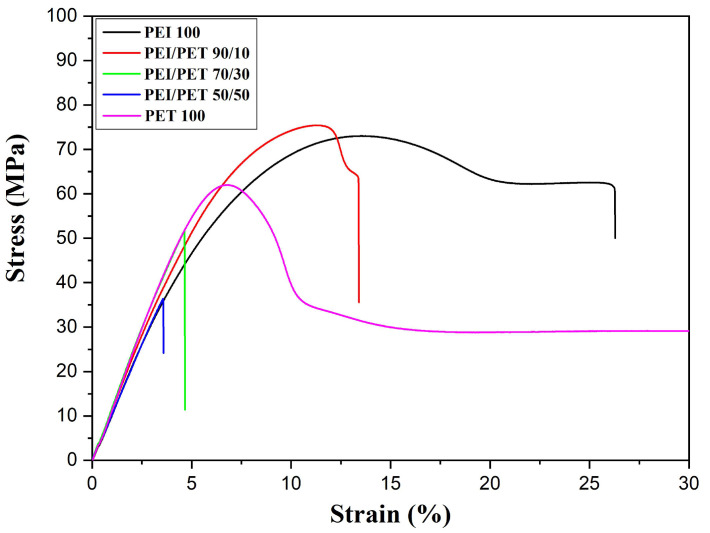
Stress–strain curve of pure PEI, pure PET, and PEI/PET polymer blends.

Polymer blends based on PEI and PET are widely used in industries requiring high mechanical strength and thermo-mechanical stability. As these blends are sensitive to structural and chemical changes, their physical and mechanical properties require in-depth analysis. The basic mechanical properties of PEI/PET blends, such as modulus of elasticity, yield strength, and elongation, were investigated during the research. Figure 7 gives the stress–strain curve of the neat PEI, PET, and PEI/PET polymer blends.

The elastic modulus (E), yield strength (σ) and strain (ε) values of the neat PEI, neat PET, and PEI/PET polymer blends are reported in Table 2. According to the mechanical test results of the study, the elastic modulus of pure PEI is ~1111 ± 30 MPa, whereas the corresponding value for pure PET is ~1158 ± 26 MPa. The modulus of elasticity initially increased with the addition of PET, peaking at 1198 ± 53 MPa for the PEI/PET 90/10 composite, demonstrating a greater stiffness than pure PEI due to the reinforcing ‘stiffening effect’ of the PET phase within the matrix. However, this trend reversed with increasing PET content, with the modulus decreasing to ~1120 ± 29 MPa in the PEI/PET 50/50 composition. Notably, the PEI/PET 70/30 blend exhibited an elastic modulus of 1177.3 ± 121 MPa; this relatively large standard deviation points to significant local variations in morphology, which may result in spatially heterogeneous stress transfer across the polymer phases [39,51]. In order to provide visual evidence for these findings, the fracture surface morphology of the PEI/PET 70/30 blend was examined via SEM (see Figure S5 in the Supplementary Information). The micrograph reveals a rugged topography characterized by hierarchical ‘hackle lines’ and multi-level fracture steps, which are indicative of a complex crack propagation path through the phase-separated polymer matrix [52]. This morphology confirms the presence of localized phase variations and micro-scale inhomogeneities, which are typical of PEI/PET systems. Such variations in the spatial distribution of the two phases explain the statistical spread observed in the elastic modulus measurements, as crack initiation and propagation are governed by these local morphological features [43,49]. As reported in the literature, the mechanical reliability of immiscible mixtures is highly sensitive to the homogeneity of stress transfer at the interface, and a fine morphology alone may not always be sufficient to ensure consistent macroscale performance [39,40]. This shows that a refined morphology alone is not sufficient to guarantee mechanical consistency in PEI/PET blends, as the lack of interfacial coupling can lead to premature failure despite a reduced dispersed phase size. Furthermore, when correlated with the quantitative AFM analysis, it becomes evident that mechanical stiffness is not directly proportional to a decrease in domain size. Although the PEI/PET 70/30 blend displays the most refined phase dispersion (562.5 nm), it does not yield the highest stiffness. The mechanical performance of PEI/PET blends depends strongly on their composition, reflecting the complex interplay between phase morphology and crystallinity. Although the yield strengths of pure PEI and PET are relatively comparable at ~73.4 ± 1.3 MPa and ~64 ± 4.4 MPa, respectively, the PEI/PET 90/10 blend maintains a high strength of 71.2 ± 9.6 MPa. This suggests that at low PET concentrations, the PEI matrix effectively incorporates the PET phase without causing significant structural disruption, which may be due to partial miscibility [51]. Conversely, the PEI/PET 50/50 blend exhibits the coarsest morphology (approximately 1 µm) and a dramatic reduction in yield strength to ~30.9 ± 4.5 MPa. This significant decline in yield strength observed in the PEI/PET 50/50 blend is likely attributed to interfacial debonding or the presence of a weak biphasic interface that acts as a stress concentrator [47,51]. Such results indicate that at intermediate compositions, insufficient interfacial adhesion compromises the structural integrity of the material, preventing effective stress transfer across polymer boundaries [51]. Furthermore, XRD results showed that while the degree of crystallinity increased with rising PET content, the elastic modulus decreased. Although crystallinity generally increases stiffness, in the PEI/PET 50/50 blend system, a higher PET content results in a phase that inherently has a lower modulus compared to the stiff PEI matrix. This observed decrease indicates that the diluting effect of low-modulus PET and the potential absence of strong interfacial bonding outweigh the reinforcing effect of the crystalline regions [47].

The elongation at break was found to be ~20 ± 6.5% for PEI and ~550 ± 48% for PET. This indicates that pure PET is highly elastic with good elongation properties. The elongation of the PEI/PET 90/10 composite was found to be ~11 ± 3.3%, and brittleness increased with the addition of PET to the blend. In the PEI/PET 50/50 composite, this decreased to 3.75 ± 0.7%. FTIR spectroscopy results showed the formation of weak hydrogen bonds between the PET carboxyl groups (C=O) and the polyether imide group, suggesting a potential contribution to the mechanical strength of blends.

### 3.6. EMI Shielding Measurements

**Figure 8:**
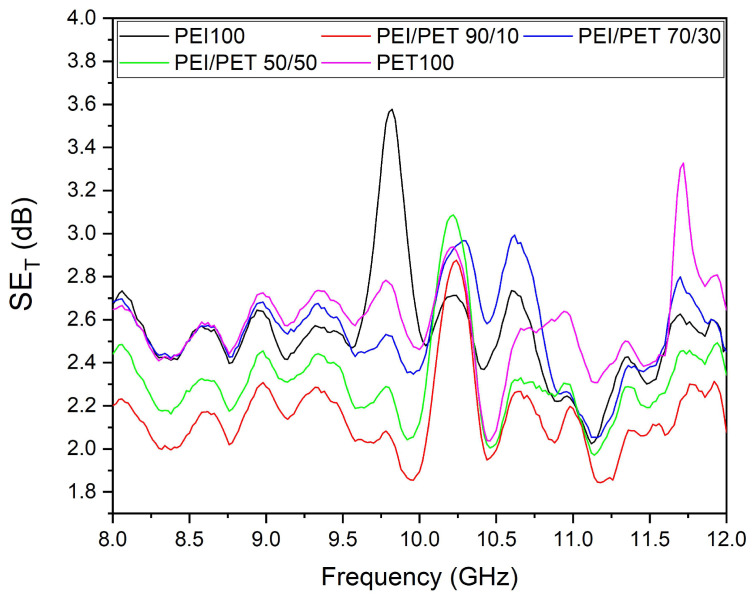
Total shielding effectiveness of neat PEI, PET, and PEI/PET polymer blends as a function of frequency over the X-band frequency range.

Based on the obtained morphology, rheological, thermal, structural, and mechanical properties, the PEI/PET polymer blends are expected to exhibit different electromagnetic shielding responses. Figure 8 shows the frequency-dependent variation in the total electromagnetic attenuation efficiency of PEI/PET polymer blends in the 8–12 GHz (X-band) range. In general, it can be seen that neat PEI, PET and PEI/PET polymer blends exhibit a slightly fluctuating behavior within the range of 1.8 to 3.6 dB depending on frequency.

Notably, the PEI/PET 90/10 composite exhibits the lowest SE_T_ values across the entire frequency range, which can be attributed to insufficient establishment of interfacial polarization due to the limited PET volume fraction and the relatively coarse domain size (928.4 ± 21.6 nm). In contrast, the PEI/PET 70/30 polymer blend displays the highest SE_T_ performance, a phenomenon directly correlated with its highly refined phase dispersion. The minimum mean domain width of 562.5 ± 12.3 nm in the PEI/PET 70/30 ratio maximizes the interfacial area, thereby enhancing electromagnetic wave attenuation through the Maxwell–Wagner–Sillars effect and increased dipolar polarization [47]. While the PEI/PET 50/50 blend has a higher PET content, it exhibits only moderate shielding efficiency; the significant domain coarsening observed in this ratio (1003 ± 27.1 nm) appears to limit the interfacial density, thus preventing the formation of an optimized polarization network required for better attenuation [13]. Furthermore, an increase in the SE_T_ values of all polymer blends in the 10–10.5 GHz frequency range has been noted. These characteristic peaks contribute to multiple internal and dipolar polarization effects within the heterogeneous polymer matrix [13].

**Figure 9:**
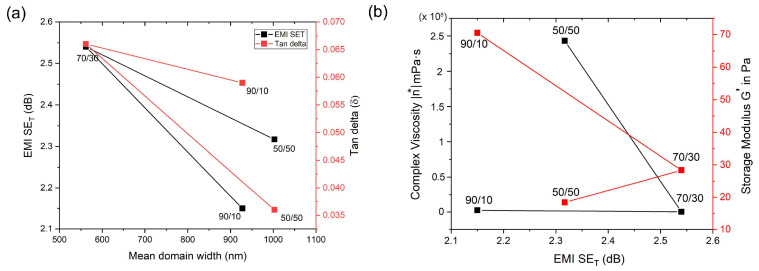
(**a**) Average EMI SE_T_ and tan δ of PEI/PET blends as a function of domain size for different blend compositions and (**b**) correlation of average EMI SE_T_ with complex viscosity and storage modulus for PEI/PET blends with different compositions.

In this study, although PEI/PET polymer blends do not contain conductive fillers that would contribute to reflection efficiency or magnetic-based fillers that would enhance absorption efficiency, they will contribute to EMI shielding efficiency in PEI/PET polymer systems that are difficult to blend, due to the mismatch in dielectric properties between the polymers. In PEI/PET systems interacting with an electromagnetic wave due to Maxwell–Wagner–Sillars (MWS) interfacial polarization, charge carriers accumulate at the interfaces between the two polymer phases, thereby contributing to EMI shielding effectiveness [53]. Consequently, the presence of phase boundaries and interfacial regions within the blend morphology plays a crucial role in promoting interfacial polarization and enhancing the dissipation of electromagnetic waves. This charge accumulation at the interfaces contributes to the dielectric loss tangent (tan δ = $\varepsilon^{\prime \prime }/\varepsilon^{\prime }$) [54], which causes the dissipation of electromagnetic waves within the material. The relationship between dielectric loss (tan δ), morphological parameters (domain size), and average total electromagnetic shielding effectiveness (SE_T_) for PEI/PET polymer blends of different compositions is shown in Figure 9a. In this case, the average SE_T_ values are regarded as the mean of the SE_T_ values determined across all frequencies. The graph clearly illustrates the effect of the morphological characteristics obtained from AFM images of PEI/PET polymer blend systems on their electromagnetic attenuation behavior.

As displayed in Figure 9a, the relationship between morphological scale and electromagnetic performance reveals a complex interplay between interfacial area and dielectric loss. The dielectric loss (tan δ) values are inversely proportional to the domain size for the studied blend compositions. The PEI/PET 50/50 blend, characterized by the coarsest morphology with a mean domain width of 1003 nm, exhibits the minimum dielectric loss (tan δ ≈ 0.036) value with a moderate SE_T_. In contrast, the PEI/PET 70/30 blend, which has the smallest mean domain width (562 ± 12.3 nm), achieves the highest average SE_T_ (2.54 dB) and dielectric loss (tan δ ≈ 0.065) due to its maximized interfacial density [47,53].

It is worth noting that the shielding values do not strictly follow the monotonic trend of the domain size. While the PEI/PET 70/30 blend achieves the highest performance, the 50/50 blend maintains a moderate shielding efficiency (2.31 ± 0.21 dB) despite having the largest domain size and the lowest tan δ values. Conversely, the PEI/PET 90/10 blend (domain width 928 nm) exhibits the minimum overall SE_T_ (2.15 ± 0.19 dB). This discrepancy suggests that, although interfacial Maxwell–Wagner–Sillars polarization is highly sensitive to domain refinement, the significantly higher volume fraction of PET in the 50/50 system provides additional attenuation mechanisms that partially compensate for the reduced interfacial density, in contrast to the PEI/PET 90/10 system, which has both a lower PET content and limited polarization density [13,47,51]. Consequently, these findings emphasize that optimizing EMI attenuation in immiscible PEI/PET systems requires a strategic balance between domain size and the specific dielectric contribution of the secondary phase [55,56]. The present results of the PEI/PET polymer blend system highlight the importance of selecting the polymer matrix when designing EMI shielding materials. The phase morphology and interfacial characteristics of the PEI/PET blend influence interfacial polarization, and are also expected to affect the localization and percolation behavior of fillers in conductive polymer composites.

The statistical significance of the EMI shielding effectiveness (SE_T_) results was evaluated to distinguish between experimental noise and true material performance. Although the maximum difference between the polymer blends is approximately 0.4 dB, the PEI/PET 70/30 blend composition consistently exhibited the highest shielding values across the 8.0–12.0 GHz range. A one-way ANOVA test was conducted on the average data, confirming that the observed variations are statistically significant *p*-value (Prob > F) < 0.05) (Figure S6) [57]. This consistency, combined with the narrow size distribution of the domains (562 ± 12.31 nm), supports the conclusion that the PEI/PET 70/30 ratio provides an optimal morphological balance for electromagnetic wave attenuation in the PEI/PET system [58].

Changes in EMI properties in polymer blends that do not contain conductive fillers (such as carbon nanotubes or graphene) and the correlation of these values with rheological properties are generally related to the reflection and scattering of EM waves from interfaces formed as a result of phase separation [59]. Furthermore, rheological parameters provide information on the viscoelastic structure of mixtures that may influence electromagnetic attenuation behavior via dielectric polarization mechanisms. Accordingly, in Figure 9b, we investigate the correlation between complex viscosity (ƞ*), storage modulus ($G^{\prime }$), and total EMI in PEI/PET blends with different compositions. Note that all complex viscosity values (ƞ*) were evaluated at a fixed frequency of 0.1 rad·s^−1^ (since the viscoelastic behavior is governed by long-range chain dynamics and microstructural interactions exist at this low frequency regime) [60] for reliable and consistent comparison among the different PEI/PET blend compositions. As seen from Figure 9b, the PEI/PET 90/10 polymer blend exhibits the highest $G^{\prime }$; this indicates that this polymer has a relatively stiff and elastic network structure due to its high PEI content. The high $G^{\prime }$ value indicates that polymer chain mobility is restricted in the PEI/PET 90/10 blend, and that there are stronger intermolecular interactions within the matrix. However, the relatively low EMI SE_T_ value (2.15 ± 0.19 dB) suggests that an extremely stiff viscoelastic structure without a conductive filler cannot significantly enhance electromagnetic attenuation alone. For the PEI/PET polymer blends, the maximum complex viscosity value (2.4 × 10^8^ mPa·s) is observed when the PET weight percentage reaches 50% (i.e., PEI/PET 50/50). This is likely due to increased interfacial interactions between the PEI and PET phases, resulting in a more interconnected morphology. The increased viscosity, coupled with stronger chain entanglement and phase interactions within the blend, appears to lead to a slight improvement in EMI shielding effectiveness, with a value of 2.31 ± 0.21 dB being reached. This is because the interfacial regions between the PEI and PET polymers contribute to the distribution of electromagnetic energy via interfacial polarization. The PEI/PET 70/30 blend has the lowest complex viscosity among all compositions and a storage modulus that is lower than that of the PEI/PET 90/10 blend, while exhibiting the highest EMI SE_T_ value (2.54 ± 0.21 dB). The reduction in rheological stiffness implies greater chain mobility and a less constrained viscoelastic network. Such a structure may facilitate greater dipole and interfacial polarization, thereby increasing dielectric loss and improving electromagnetic wave attenuation. The higher dielectric loss tangent value shown in Figure 9a compared to other polymer blends also supports the relationship between rheological properties and EMI.

## 4. Conclusions

The present study provides detailed information on the structural, morphological, rheological, mechanical, and thermal behavior of twin screw extruded PEI/PET polymer blends with different compositions (90/10, 70/30, and 50/50 *w*/*w*%). In addition, the correlations between the nanoscale morphologies, viscoelastic behavior, and electromagnetic response in the filler-free PEI/PET system have been investigated. FTIR results have demonstrated that molecular interactions between polymer components influence their physicochemical properties. In particular, the splitting and shifts in the absorption bands of carbonyl groups indicate varying degrees of interaction between these groups. The vibrational bands of the aromatic rings and methyl groups also change, confirming the presence of steric effects. Quantitative atomic force microscopy (AFM) results confirm a nonlinear dependency of the blend morphology on the component ratio. The PEI/PET 70/30 blend achieves the most refined phase dispersion, with a minimum mean domain width of 562.5 nm. By contrast, significant morphological coarsening is observed when the composition deviates from this ratio, with domain widths increasing to 928.4 nm in the 90/10 blend and reaching a maximum of around 1 µm in the PEI/PET 50/50 blend. XRD analysis indicates that all PEI/PET blends predominantly exhibit amorphous characteristics. A broad halo confirms the absence of long-range order, while variations in PET content induce shifts in peak position and intensity, reflecting changes in chain packing. Tensile testing revealed that PEI and PET blends exhibited high mechanical strength in terms of both elastic modulus and yield strength. The mechanical results showed that incorporating a small amount of PET (90/10) preserved the tensile strength of PEI, whereas a higher content of PET (50/50) led to a noticeable reduction in yield strength. The immiscibility of the PEI/PET blends, initially indicated by the two-step degradation in TGA and phase-separated morphologies in AFM, is further corroborated by the DMA results. Specifically, the emergence of asymmetric shoulders and broadened transition regions in the loss factor profiles signifies a heterogeneous relaxation spectrum, confirming an immiscible phase structure where PET segments coexist in both bulk-like environments and interfacially constrained regions under the influence of the matrix confinement effect. Consequently, the structural refinement observed in the PEI/PET 70/30 composition provides the optimal interfacial area required to maximize Maxwell–Wagner–Sillars polarization, whereas the coarser phase distribution in the 50/50 and 90/10 ratios moderates the further enhancement of interfacial polarization and subsequent shielding effectiveness.

The findings of this study provide a fundamental framework for understanding the role of immiscible polymer blend morphologies in EMI applications. The maximum EMI shielding effectiveness of 2.54 dB obtained in this study serves to validate the concept of interface-induced attenuation rather than serving as a practical or definitive shielding material; however, it highlights the importance of Maxwell–Wagner–Sillars polarization at phase boundaries. Such phase-separated structures are known to facilitate the formation of conductive networks at low percolation thresholds through the selective localization of additives, which can significantly enhance electrical conductivity and EMI performance. In this regard, blends incorporating high-temperature engineering polymers, such as PEI, offer significant potential for EMI applications by enabling effective matrix selection and design. Further studies build upon this foundation by incorporating conductive fillers, such as graphene, carbon black, and Ag NWs, and magnetic fillers, such as Fe_3_O_4_, into optimized PEI/PET blend morphologies to elevate the electromagnetic performance to practical shielding thresholds.

## Figures and Tables

**Table 1:** Melt flow index values of the polymer blends, measured according to ISO1133 under PEI-specific testing conditions.

Sample	MFI	ISO1133
PEI 100	65	5 kg/340 °C
PEI/PET 90/10	124	5 kg/340 °C
PEI/PET 70/30	312	5 kg/340 °C
PET 100	69	2.16 kg */280 °C

* Note: PET 100 was measured at its standard characterization conditions (280 °C/2.16 kg). Due to the risk of rapid thermal degradation and excessive flow at 340 °C, pure PET could not be reliably tested under the same conditions as the PEI blends.

**Table 2:** Mechanical properties of polymer blends.

Sample	E, [MPa]	σ, [MPa]	ε, [%]
PET100	1157.6 ± 26	63.95 ± 4.41	548.91 ± 47.3
PEI/PET 50/50	1119.9 ± 29	30.86 ± 4.45	3.75 ± 0.67
PEI/PET 70/30	1177.3 ± 121	46.97 ± 10.84	4.41 ± 1.21
PEI/PET 90/10	1198.4 ± 53	71.22 ± 9.60	11.14 ± 3.34
PEI100	1110.87 ± 30	73.38 ± 1.3	20.14 ± 6.4

## Data Availability

The original contributions presented in this study are included in the article/Supplementary Material. Further inquiries can be directed to the corresponding author.

## Supplementary Materials

The following supporting information can be downloaded at: https://www.mdpi.com/article/10.3390/polym18121528/s1, Figure S1. XRD pattern deconvolution and Lorentz fitting results for the PEI/PET (70:30) blend. The deconvolution distinguishes the amorphous halo from the specific triclinic crystalline reflections of PET [35,36]. The accompanying table details the refined peak parameters, showing an excellent fit with an adjusted R^2^ of 0.99585, which was used for the subsequent triclinic unit cell calculations [37]. Figure S2. AFM phase images of (a) PEI/PET 90/10, (b) PEI/PET 70/30, and (c) PEI/PET 50/50 polymer blends. Figure S3. The statistical distribution of the PET domain widths. Figure S4. DSC thermograms of neat PET (recycled PET) and PEI polymers (heating rate: 10 °C/min). Figure S5. SEM micrograph (2000×) showing the fracture surface of the PEI/PET 70/30 blend. The rugged topography and the presence of ‘hackle lines’ provide visual confirmation of the structural heterogeneity and phase separation that contribute to the observed variation in mechanical properties [52]. Figure S6. Comparative analysis of Electromagnetic Interference Shielding Effectiveness for PEI/PET blends across different ratios: Group C (90/10) with a value of 2.15 dB, Group D (70/30) with 2.54 dB, and Group E (50/50) with 2.31 dB. Data are presented as mean ± standard deviation with corresponding ranges for each experimental group. One-way ANOVA indicates statistically significant differences in shielding performance across all samples (*p*-value (Prob > F) <0.05).

## Abbreviations

The following abbreviations are used in this manuscript:

PEI	Polyetherimide
PET	Poly(ethylene terephthalate)
EMI	Electromagnetic inference
SE	Shielding efficiency
AFM	Atomic force microscopy
DMA	Dynamic mechanical analysis
TGA	Thermogravimetric analysis
XRD	X-ray diffraction
FTIR	Fourier transform infrared spectroscopy
