# Supplementary material for: Structure–Property Relationships in PEI/PET Polymer Blends: Morphological, Rheological, Thermal, Mechanical Behavior, and Electromagnetic Response
Source: Polymers (Basel). 2026 Jun 19;18(12):1528. doi: 10.3390/polym18121528 (PMC13306277; doi:10.3390/polym18121528)
Supplement: Supplementary file 1 [file polymers-18-01528-s001.zip › polymers-4350609-supplementary.pdf]

Supplementary Information

Structure-Property Relationships in PEI/PET Polymer Blends: Morphological, Rheological, Thermal, Mechanical Behavior, and Electromagnetic Response

Elshod Olmosovich Khakberdiev[1], Hülya Kaftelen Odabaşı[2]*, Akın Odabaşı[3], Selcuk Helhel[4], Qodirbek Nuridin ugli Berdinazarov[1], Nizomiddin Zokir ugli Dusiyorov[1], Nigmat Rustamovich Ashurov[1]

[1]Institute of Polymer Chemistry and Physics, Uzbekistan Academy of Sciences, Tashkent Uzbekistan; profhaqberdiyev@gmail.com, qodirberdinazarov@gmail.com, ndusiyorov@gmail.com, nigmat.ashurov@gmail.com
[2*]School of Civil Aviation, Dept. of Aircraft Maintenance and Repair, Fırat University, 23200 Elazığ, Turkey; hkodabasi@firat.edu.tr

[3]Engineering Faculty, Dept. of Metallurgical and Materials Engineering, Fırat University, 23200 Elazığ, Turkey; odabasia@firat.edu.tr

[4]Engineering Faculty, Dept. of Electrical & Electronics Engineering, Akdeniz University, 07058 Antalya, Turkey; selcukhelhel@akdeniz.edu.tr

*Correspondence: hkodabasi@firat.edu.tr

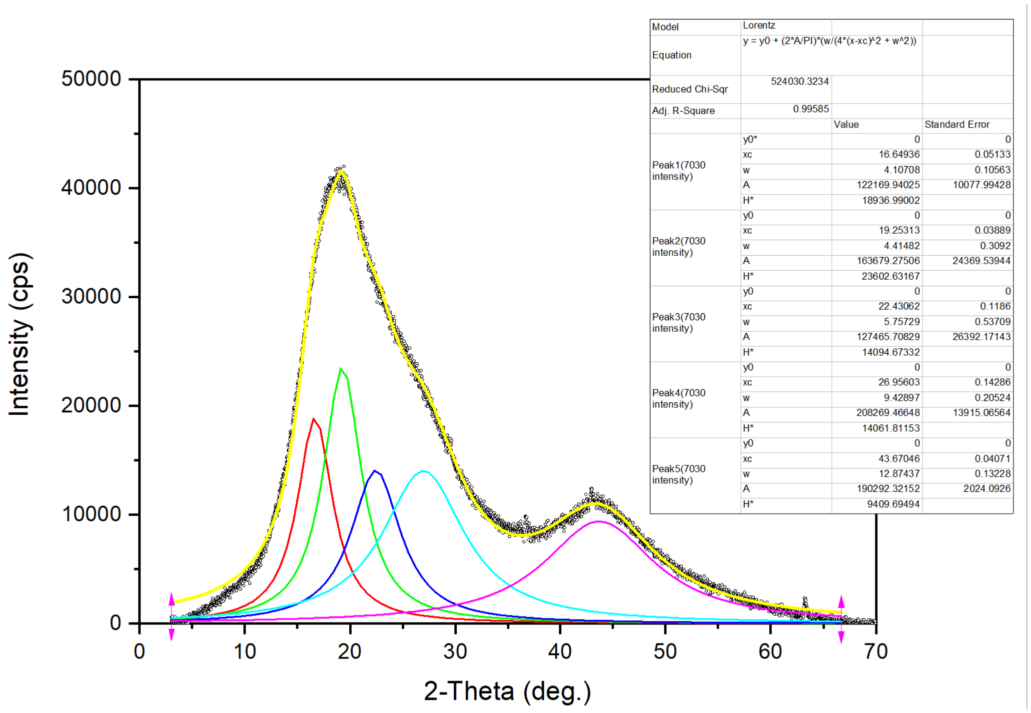

Model	Lorentz		
Equation	y = y0 + (2*A/PI)*(w/(4*(x-xc)^2 + w^2))		
Reduced Chi-Sqr	524030.3234		
Adj. R-Square	0.99585		
		Value	Standard Error
Peak1(7030 intensity)	y0*	0	0
	xc	16.64936	0.05133
	w	4.10708	0.10563
	A	122169.94025	10077.99428
	H*	18936.99002	
Peak2(7030 intensity)	y0	0	0
	xc	19.25313	0.03889
	w	4.41482	0.3092
	A	163679.27506	24369.53944
	H*	23602.63167	
Peak3(7030 intensity)	y0	0	0
	xc	22.43062	0.1186
	w	5.75729	0.53709
	A	127465.70829	26392.17143
	H*	14094.67332	
Peak4(7030 intensity)	y0	0	0
	xc	26.95603	0.14286
	w	9.42897	0.20524
	A	208269.46648	13915.06564
	H*	14061.81153	
Peak5(7030 intensity)	y0	0	0
	xc	43.67046	0.04071
	w	12.87437	0.13228
	A	190292.32152	2024.0926
	H*	9409.69494	

Figure S1. XRD pattern deconvolution and Lorentz fitting results for the PEI/PET (70:30) blend. The deconvolution distinguishes the amorphous halo from the specific triclinic crystalline reflections of PET [35, 36]. The accompanying table details the refined peak parameters, showing an excellent fit with an adjusted R^2 of 0.99585, which was used for the subsequent triclinic unit cell calculations [37].

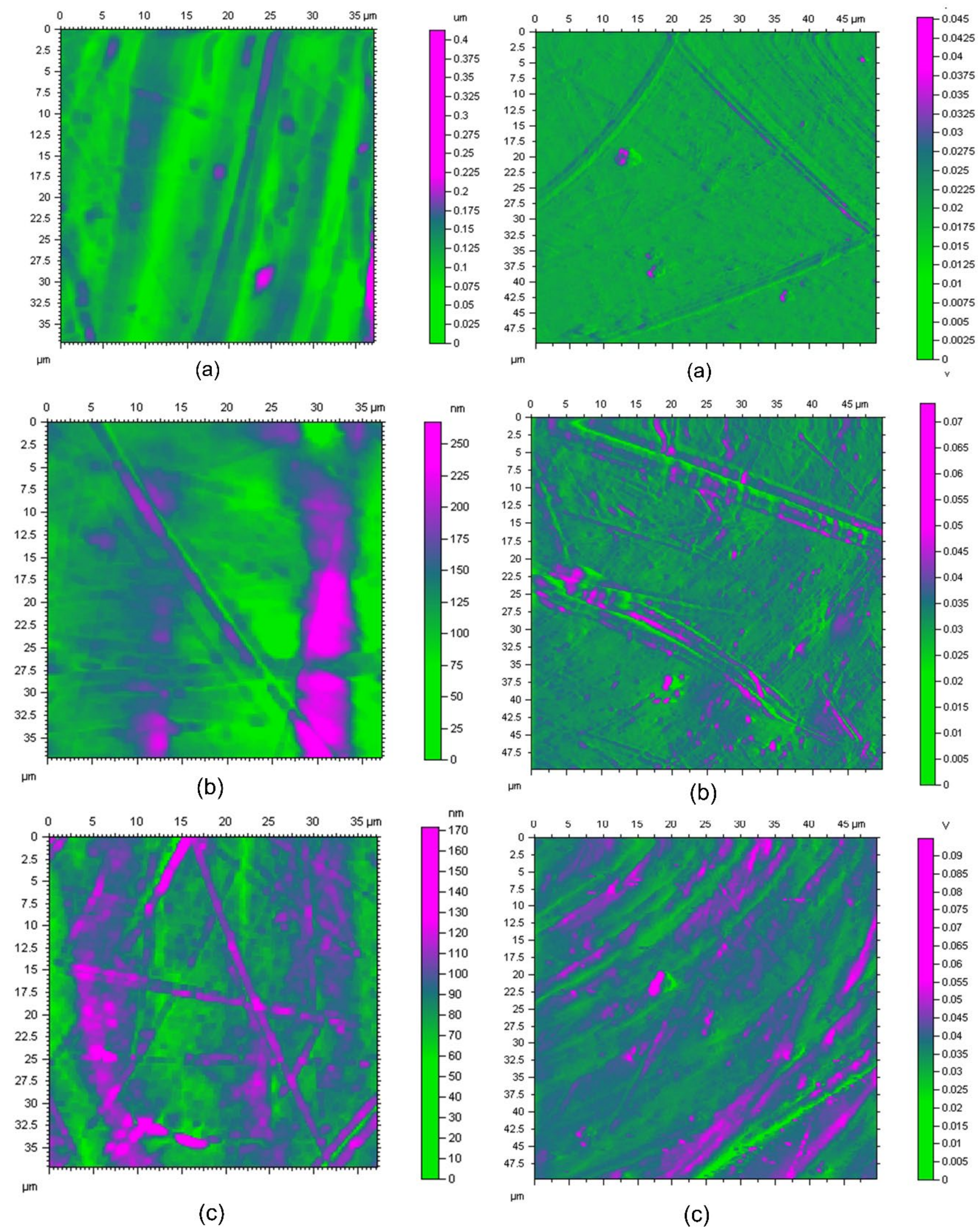

Figure S2. AFM phase images of (a) PEI/PET 90/10,(b) PEI/PET 70/30 and (c) PEI/PET 50/50 polymer blends.

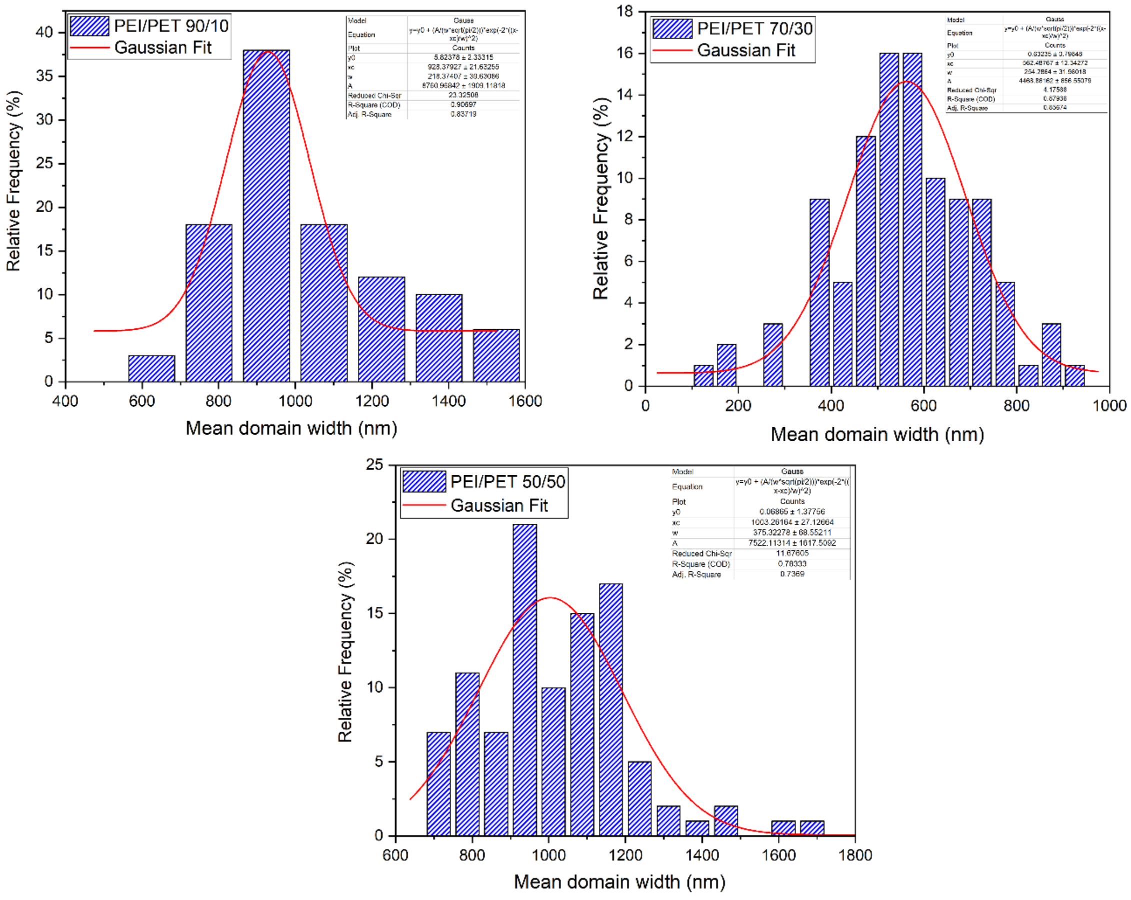

Figure S3. The statistical distribution of the PET domain widths.

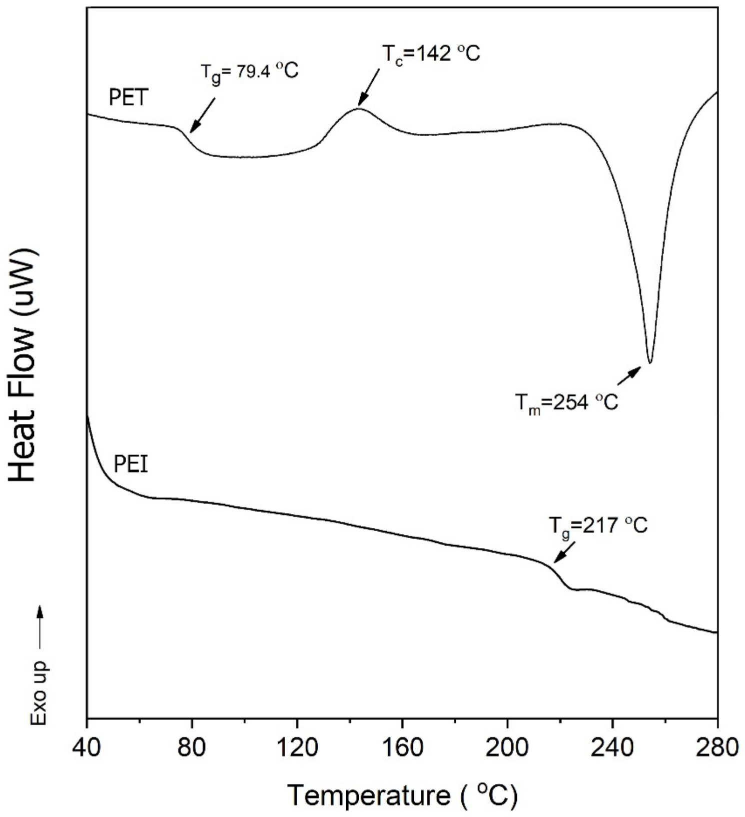

Figure S4. DSC thermograms of neat PET (recycled PET) and PEI polymers (heating rate: 10 °C/min).

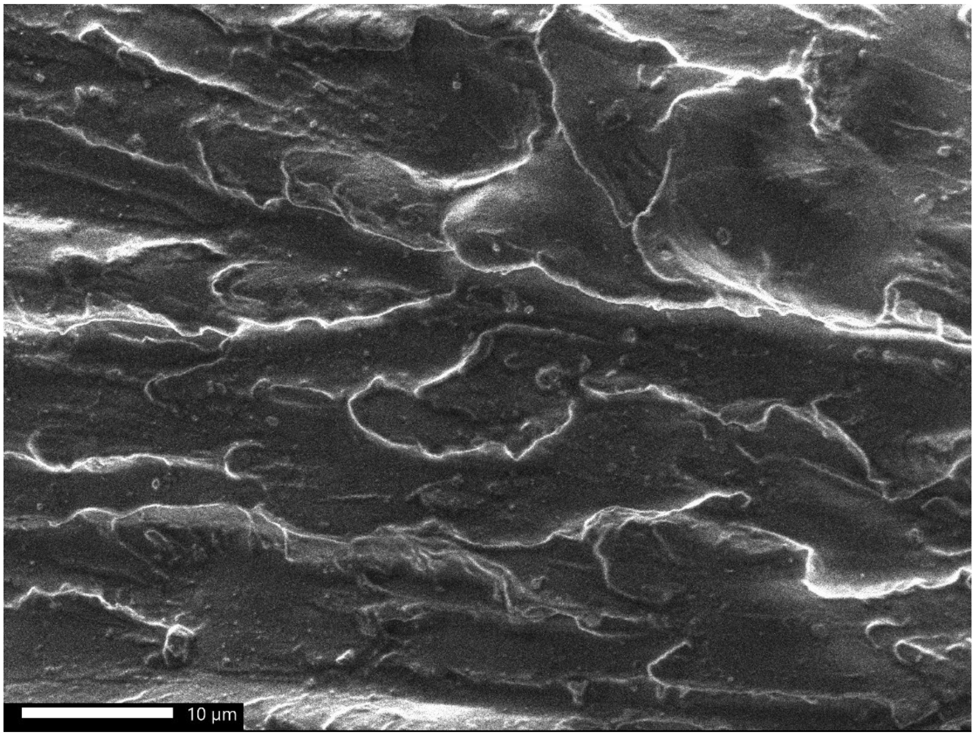

Figure S5. SEM micrograph (2kX) showing the fracture surface of the PEI/PET 70/30 blend. The rugged topography and the presence of 'hackle lines' provide visual confirmation of the structural heterogeneity and phase separation that contribute to the observed variation in mechanical properties [52].

ANOVAOneWay (4/29/2026 22:04:30)

Descriptive Statistics

	N Analysis	N Missing	Mean	Standard Deviation	SE of Mean
C	202	2	2.1502	0.18555	0.01306
D	202	1	2.54186	0.2014	0.01417
E	202	1	2.31658	0.20097	0.01414

One Way ANOVA

Overall ANOVA

	DF	Sum of Squares	Mean Square	F Value	Prob>F
Model	2	15.60958	7.80479	202.92908	0
Error	603	23.1918	0.03846		
Total	605	38.80138			

Null Hypothesis: The means of all levels are equal.
Alternative Hypothesis: The means of one or more levels are different.
At the 0.05 level, the population means are significantly different.

Fit Statistics

	R-Square	Coeff Var	Root MSE	Data Mean
	0.40229	0.08395	0.19611	2.33621

Figure S6. Comparative analysis of Electromagnetic Interference Shielding Effectiveness for PEI/PET blends across different ratios: Group C (90/10) with a value of 2.15dB, Group D (70/30) with 2.54dB, and Group E (50/50) with 2.31dB. Data are presented as mean ± standard deviation with corresponding ranges for each experimental group. One-way ANOVA indicates statistically significant differences in shielding performance across all samples (p value (Prob>F) <0.05).
